# Type VI secretion systems of human gut Bacteroidales segregate into three genetic architectures, two of which are contained on mobile genetic elements

**DOI:** 10.1186/s12864-016-2377-z

**Published:** 2016-01-15

**Authors:** Michael J. Coyne, Kevin G. Roelofs, Laurie E. Comstock

**Affiliations:** Division of Infectious Diseases, Brigham and Women’s Hospital, Harvard Medical School, 181 Longwood Avenue, Boston, MA 02115 USA

**Keywords:** Type VI secretion system, T6SS, ICE – integrative and conjugative element, Bacteroidales, Gut microbiota

## Abstract

**Background:**

Type VI secretion systems (T6SSs) are contact-dependent antagonistic systems employed by Gram negative bacteria to intoxicate other bacteria or eukaryotic cells. T6SSs were recently discovered in a few Bacteroidetes strains, thereby extending the presence of these systems beyond Proteobacteria. The present study was designed to analyze in a global nature the diversity, abundance, and properties of T6SSs in the Bacteroidales, the most predominant Gram negative bacterial order of the human gut.

**Results:**

By performing extensive bioinformatics analyses and creating hidden Markov models for Bacteroidales Tss proteins, we identified 130 T6SS loci in 205 human gut Bacteroidales genomes. Of the 13 core T6SS proteins of Proteobacteria, human gut Bacteroidales T6SS loci encode orthologs of nine, and an additional five other core proteins not present in Proteobacterial T6SSs. The Bacteroidales T6SS loci segregate into three distinct genetic architectures with extensive DNA identity between loci of a given genetic architecture. We found that divergent DNA regions of a genetic architecture encode numerous types of effector and immunity proteins and likely include new classes of these proteins. TheT6SS loci of genetic architecture 1 are contained on highly similar integrative conjugative elements (ICEs), as are the T6SS loci of genetic architecture 2, whereas the T6SS loci of genetic architecture 3 are not and are confined to *Bacteroides fragilis.* Using collections of co-resident Bacteroidales strains from human subjects, we provide evidence for the transfer of genetic architecture 1 T6SS loci among co-resident Bacteroidales species in the human gut. However, we also found that established ecosystems can harbor strains with distinct T6SS of all genetic architectures.

**Conclusions:**

This is the first study to comprehensively analyze of the presence and diversity of T6SS loci within an order of bacteria and to analyze T6SSs of bacteria from a natural community. These studies demonstrate that more than half of our gut Bacteroidales, equivalent to about ¼ of the bacteria of this ecosystem, encode T6SSs. The data reveal several novel properties of these systems and suggest that antagonism between or distributed defense among these abundant intestinal bacteria may be common in established human gut communities.

**Electronic supplementary material:**

The online version of this article (doi:10.1186/s12864-016-2377-z) contains supplementary material, which is available to authorized users.

## Background

The human intestinal microbiota contains more than 35 species of the order Bacteroidales that are collectively the most abundant Gram negative bacteria of this ecosystem. These species co-colonize the human intestine at very high density [1–4] and form stable ecosystems [4], with strains persisting for years or decades. The co-residence, high abundance, and long term colonization by the Bacteroidales suggest that they may form mutualistic relationships that stabilize the community. In addition, as newly introduced strains are rarely able to become established, there are likely antagonistic mechanisms employed by these strains to compete and defend the ecosystem from invasion. Relatively few studies have analyzed interactions between predominant human gut Bacteroidales strains. Gut Bacteroidales are equipped with large arsenals of proteins that allow them to harvest a tremendous array of different plant polysaccharides and host-derived glycans (reviewed [5]). We have shown that Bacteroidales strains form complex ecological networks based on the breakdown and utilization of dietary polysaccharides, such that some strains that cannot access a certain polysaccharide can be cross-fed by other Bacteroidales strains able to utilize the polysaccharide [6]. These interactions benefit at least one interacting member, likely without harm to either partner. We have also shown that competitive interactions via direct antagonism occur among Bacteroidales strains. Gut Bacteroidales secrete antibacterial proteins, designated BSAPs, one of which we have shown does not require a cognate immunity protein and targets closely related strains [7]. As bacteria use many different mechanisms to compete in microbial communities, and the gut microbiota is an extremely dense ecosystem, we predicted that contact dependent Type VI secretion systems (T6SSs) are likely very prevalent antagonistic systems of the gut Bacteroidales.

T6SSs are toxin delivery systems that until recently were only described in Proteobacterial species. The T6SS apparatus is a multiprotein complex requiring numerous core proteins (Tss proteins) including cytoplasmic, transmembrane, and outer membrane components [8, 9]. The needle or tube apparatus is comprised of a phage-like complex, similar to the T4 contractile bacteriophage tail, which is thought to be anchored to the membrane by a trans-envelope complex. These tube and trans-envelope sub-assemblies are linked via TssK [10]. The inner tube is comprised of Hcp (TssD) that assemble as stacked hexamers [11]. The VgrG (TssI) protein sits at the tip of the tube and forms a spike (in some cases, sharpened by a PAAR protein [12]), which enables puncturing of the recipient’s membranes. Toxic effectors are loaded onto the tube/spike apparatus by interacting with Hcp or VgrG [13–15]. The inner tube is surrounded by a sheath comprised of TssB and TssC that contracts [16, 17], driving expulsion of the inner tube from the cell. Current models suggest that this T6SS firing delivers the contents of the inner tube either into the supernatant of *in vitro* grown bacteria, or punctures the membrane of a neighboring bacterial or eukaryotic cell thereby delivering the toxic contents. Following T6SS firing, components of the T6SS machinery are recycled using energy provided by the ATPase ClpV (TssH) [18, 19]. T6SS effectors include cell wall degrading enzymes, proteins that affect cell membranes (phospholipases, pore-forming), and nucleases [20]. In most cases, the effector is produced with a cognate immunity protein, typically encoded by an adjacent gene [21]. Both eukaryotic and bacterial cells are targeted by T6SS effectors (reviewed [22]) although most described T6SS effectors target Gram negative bacteria.

T6SSs were only recently identified in Bacteroidetes species [23–25] and this lapse was due to the fact that many proteins of the few identified Bacteroidetes T6SS do not share sufficient sequence similarity with core T6SS proteins of Proteobacteria to be detectable by methods that rely on protein-protein comparisons (e.g. blastp) or protein-profile comparisons (e.g. Pfam, COG). Using more sensitive profile-profile and profile-structural comparisons, we identified a T6SS locus contained on an integrative conjugative element (ICE), and showed that it was transferred between four different Bacteroidales species while co-resident in a human gut [23]. This was the first demonstration of a T6SS locus being transferred between members of a natural microbial community. A concurrent study showed functional T6SSs in two Bacteroidetes species [24]. The soil organism *Flavobacterium johnsonii* was shown to have T6SS proteins that function analogously to their Proteobacterial counterparts, and an effector and its cognate immunity protein were identified. In addition, a T6SS of *Bacteroides fragilis* was shown to antagonize a *Bacteroides thetaiotaomicron* strain *in vitro* [24]. The Bacteroidetes T6SSs are distinct enough from the general Proteobacterial T6SS loci (T6SS^i^) and the *Francisella* T6SSs (T6SS^ii^) that they have been designated a separate subtype (T6SS^iii^) (24). In the present study, we build upon these early findings and provide a comprehensive analysis of the prevalence and properties of T6SS loci of the human gut Bacteroidales.

## Results

### Identification of T6SS loci in human gut Bacteroidales strains

In order to perform a comprehensive analysis of T6SSs of human gut Bacteroidales, we retrieved the genome sequences of available human gut Bacteroidales strains deposited to Genbank and Refseq comprising a total of 205 strains with draft and complete genome sequences representing 35 different species (Table 1). As some strains were identified only to the genus level, the 16S rRNA sequences were retrieved for all strains and each was assigned a species designation based on the closest match in the ribosomal database (Table 1). We took advantage of the fact that the genes encoding most of the proteins involved in T6S are clustered in a T6SS locus. The protein sequences of these 205 genomes were searched for two proteins, VgrG (TssI) and ClpV (TssH), which are consistently present in T6SSs and are two of the few Bacteroidetes T6SS proteins we previously found to be identifiable by sequence profile searches using profiles generated from their Proteobacterial counterparts. If genes encoding proteins containing motifs TIGR03361 (VgrG) and TIGR03345 (ClpV) were found on the same contig or scaffold and were within fifteen genes of each other, all intervening genes and 25 genes flanking each side were retrieved. This search identified a total of 115 regions from 102 of the 205 strains and included 16 different *Bacteroides* and *Parabacteroides* species (Table 1). All the proteins encoded by these 115 regions were clustered using cut-offs of 30 % identity over at least 70 % of the protein, and a representative protein from each cluster was searched for motifs (using traditional sequence-profile searches) and further analyzed by profile-profile and profile-structural comparisons. Many of these T6SS proteins could only be identified using the more sensitive profile-profile and profile-structural comparisons. Table 2 shows the cluster analysis of the protein families identified most frequently from these 115 regions with corresponding hits to various databases. These 115 regions were then trimmed in a consistent manner to contain all identified *tss* genes as well as genes encoding putative TetR/AcrR family transcriptional regulators, T6SS-associated genes (Tags) described below, and genes encoding predicted effector and/or immunity proteins. The trimming of these segments, although consistent, was somewhat arbitrary and is our best prediction for the boundaries of these regions. All 115 regions identified in the initial search for VgrG and ClpV were found to contain multiple additional Tss encoding genes, with most regions containing genes encoding TssB, TssC, Hcp, TssE, TssF, and TssG. Regions lacking this full complement of Tss encoding genes were present on contigs that terminate within the T6SS locus, and for some of these regions, the remainder of these genes were identified on other contigs (Additional file 1, marked as “extension”). The identified T6SS loci are distributed among *Bacteroides* and *Parabacteroides* genomes with none of the nine *Alistipes* genomes containing a T6SS identified by these methods. To ensure the comprehensive nature of this search, we created Hidden Markov Model (HMM) profiles of all proteins with Tss, Tag, TetR, PAAR or Rhs designations and used them to search the 205 genomes again for matches. For example, in these 115 regions there are 400 predicted genes encoding Hcp proteins, which segregate into 11 distinct clusters (Table 2 and Additional file 2). The sequences of these 400 proteins were used to make a profile HMM for the Bacteroidales Hcp family. We used the resulting profiles of all Tss, Tag, TetR, PAAR or Rhs families to query the protein sequences of the full genome set and identified 15 additional T6SS loci (Table 1, Additional file 1). These regions were not identified in the initial analysis most commonly because the VgrG and ClpV proteins are encoded by genes on different contigs, a consequence of the incomplete nature of many of these genome sequences. In addition to *Bacteroides* and *Parabacteroides*, these new analyses identified a putative T6SS in *Prevotella stercorea* DSM 18206. This analysis not only identified all T6SS loci present in these genomes, but also any gene encoding a Tss protein that is not contained within these T6SS loci. Numerous ClpV-like encoding genes were identified outside of T6SS regions, which is not surprising considering that ClpV is an ATPase. In addition, Rhs proteins and transcriptional regulators of the TetR family were also frequently identified outside T6SS regions, and occasionally Hcp and VgrG proteins, also components of phage, were infrequently found to be encoded outside T6SS regions (Additional file 1).

**Table 1:** Summary of the 205 human gut Bacterodales strains analyzed and the T6SS loci present in each genome

					T6SS architecture				
number	genome	16S ID	NCBI Bioproject ID	loci initially identified	1	2	3	other	identified by HMM
1	*Alistipes finegoldii* DSM 17242	*A. finegoldii*	PRJNA40775	0					
2	*Alistipes indistinctus* YIT 12060	*A. indistinctus*	PRJNA46373	0					
3	*Alistipes onderdonkii* DSM 19147	*A. onderdonkii*	PRJNA199292	0					
4	*Alistipes putredinis* DSM 17216	*A. putredinis*	PRJNA19655	0					
5	*Alistipes senegalensis* JC50	*A. senegalensis*	PRJNA199660	0					
6	*Alistipes shahii* WAL 8301	*A. shahii*	PRJNA45913	0					
7	*Alistipes sp.* AL-1	*A. onderdonkii*	PRJNA224116	0					
8	*Alistipes sp.* HGB5	*A. finegoldii*	PRJNA54031	0					
9	*Alistipes timonensis* JC136	*A. timonensis*	PRJEA174622	0					
10	*Bacteroides caccae* ATCC 43185	*B. caccae*	PRJNA18163	0					
11	*Bacteroides caccae* CL03T12C61	*B. caccae*	PRJNA64801	1		1			
12	*Bacteroides cellulosilyticus* CL02T12C19	*B. cellulosilyticus*	PRJNA64803	1	1				
13	*Bacteroides cellulosilyticus* DSM 14838	*B. cellulosilyticus*	PRJNA30027	0					
14	*Bacteroides cellulosilyticus* WH2	*B. cellulosilyticus*	PRJNA224116	0					
15	*Bacteroides sp.* 14(A)	*B. cellulosilyticus*	PRJNA224116	0					
16	*Bacteroides clarus* YIT 12056	*B. clarus*	PRJNA48509	0					
17	*Bacteroides coprocola* DSM 17136	*B. coprocola*	PRJNA20521	0					
18	*Bacteroides coprophilus* DSM 18228	*B. coprophilus*	PRJNA30371	1	1				1
19	*Bacteroides dorei* 5 1 36-D4	*B. dorei*	PRJNA32451	0					
20	*Bacteroides dorei* CL02T00C15	*B. dorei*	PRJNA64805	1	1				
21	*Bacteroides dorei* CL02T12C06	*B. dorei*	PRJNA64807	1	1				
22	*Bacteroides dorei* CL03T12C01	*B. dorei*	PRJNA64809	0					
23	*Bacteroides dorei* DSM 17855	*B. dorei*	PRJNA27831	1		1			
24	*Bacteroides dorei* HS1 L 1 B 010	*B. dorei*	PRJNA232731	1	1				
25	*Bacteroides dorei* HS1 L 3 B 079	*B. dorei*	PRJNA232731	0					
26	*Bacteroides sp.* 3 1 33FAA	*B. dorei*	PRJNA38353	0					
27	*Bacteroides sp.* 9 1 42FAA	*B. dorei*	PRJNA32445	0					
28	*Bacteroides eggerthii* 1 2 48FAA	*B. eggerthii*	PRJNA40009	0					
29	*Bacteroides eggerthii* DSM 20697	*B. eggerthii*	PRJNA27827	1		1			
30	*Bacteroides faecis* MAJ27	*B. faecis*	PRJNA86875	0					
31	*Bacteroides finegoldii* CL09T03C10	*B. finegoldii*	PRJNA64831	1	1				
32	*Bacteroides finegoldii* DSM 17565	*B. finegoldii*	PRJNA27823	0					
33	*Bacteroides fluxus* YIT 12057	*B. fluxus*	PRJNA48511	1				1	
34	*Bacteroides fragilis* 3 1 12	*B. fragilis*	PRJNA32433	1	1				
35	*Bacteroides fragilis* 638R	*B. fragilis*	PRJNA50405	1			1		
36	*Bacteroides fragilis* CL03T00C08	*B. fragilis*	PRJNA64811	1			1		
37	*Bacteroides fragilis* CL03T12C07	*B. fragilis*	PRJNA64813	1			1		
38	*Bacteroides fragilis* CL05T00C42	*B. fragilis*	PRJNA64815	1		1			
39	*Bacteroides fragilis* CL05T12C13	*B. fragilis*	PRJNA64817	1		1			
40	*Bacteroides fragilis* CL07T00C01	*B. fragilis*	PRJNA64819	1			1		
41	*Bacteroides fragilis* CL07T12C05	*B. fragilis*	PRJNA64821	1			1		
42	*Bacteroides fragilis* DCMOUH0017B	*B. fragilis*	PRJNA244943	2			2		
43	*Bacteroides fragilis* DCMOUH0018B	*B. fragilis*	PRJNA244944	0					
44	*Bacteroides fragilis* DCMOUH0042B	*B. fragilis*	PRJNA253771	1			1		
45	*Bacteroides fragilis* DCMOUH0067B	*B. fragilis*	PRJNA254401	0					
46	*Bacteroides fragilis* DCMOUH0085B	*B. fragilis*	PRJNA254455	1			1		
47	*Bacteroides fragilis* DCMSKEJBY0001B	*B. fragilis*	PRJNA244942	0					
48	*Bacteroides fragilis* HMW 610	*B. fragilis*	PRJNA71525	0					
49	*Bacteroides fragilis* HMW 615	*B. fragilis*	PRJNA71527	0					
50	*Bacteroides fragilis* HMW 616	*B. fragilis*	PRJNA71529	1			1		
51	*Bacteroides fragilis* JCM 11017	*B. fragilis*	PRJNA224116	0					1
52	*Bacteroides fragilis* NCTC 9343	*B. fragilis*	PRJNA46	1			1		
53	*Bacteroides fragilis* YCH46	*B. fragilis*	PRJNA13067	2	1		1		
54	*Bacteroides fragilis* str 1007-1-F #10	*B. fragilis*	PRJNA206138	1			1		
55	*Bacteroides fragilis* str 1007-1-F #3	*B. fragilis*	PRJNA206180	1			1		
56	*Bacteroides fragilis* str 1007-1-F #4	*B. fragilis*	PRJNA206181	1			1		
57	*Bacteroides fragilis* str 1007-1-F #5	*B. fragilis*	PRJNA206182	1			1		
58	*Bacteroides fragilis* str 1007-1-F #6	*B. fragilis*	PRJNA206183	1			1		
59	*Bacteroides fragilis* str 1007-1-F #7	*B. fragilis*	PRJNA206135	1			1		
60	*Bacteroides fragilis* str 1007-1-F #8	*B. fragilis*	PRJNA206136	1			1		
61	*Bacteroides fragilis* str 1007-1-F #9	*B. fragilis*	PRJNA206137	1			1		
62	*Bacteroides fragilis* str 1009-4-F #10	*B. fragilis*	PRJNA206140	1			1		
63	*Bacteroides fragilis* str 1009-4-F #7	*B. fragilis*	PRJNA206139	1			1		
64	*Bacteroides fragilis* str 2-F-2 #4	*B. fragilis*	PRJNA206111	1	1				
65	*Bacteroides fragilis* str 2-F-2 #5	*B. fragilis*	PRJNA206112	1	1				
66	*Bacteroides fragilis* str 2-F-2 #7	*B. fragilis*	PRJNA206113	1	1				
67	*Bacteroides fragilis* str 20793-3	*B. fragilis*	PRJNA206110	0					
68	*Bacteroides fragilis* str 3-F-2 #6	no call	PRJNA206178	0					1
69	*Bacteroides fragilis* str 3397 N2	*B. fragilis*	PRJNA206143	0					
70	*Bacteroides fragilis* str 3397 N3	*B. fragilis*	PRJNA206144	0					
71	*Bacteroides fragilis* str 3397 T10	*B. xylanisolvens*	PRJNA206115	0					
72	*Bacteroides fragilis* str 3397 T14	*B. fragilis*	PRJNA206142	0					
73	*Bacteroides fragilis* str 34-F-2 #13	*B. fragilis*	PRJNA206179	1	1				
74	*Bacteroides fragilis* str 3719 A10	*B. fragilis*	PRJNA206150	0					
75	*Bacteroides fragilis* str 3719 T6	*B. fragilis*	PRJNA206149	0					
76	*Bacteroides fragilis* str 3725 D9(v)	*B. fragilis*	PRJNA206141	2	1		1		
77	*Bacteroides fragilis* str 3725 D9 ii	*B. ovatus*	PRJNA206117	0					
78	*Bacteroides fragilis* str 3774 T13	*B. fragilis*	PRJNA206151	2	1		1		
79	*Bacteroides fragilis* str 3783 N1-2	*B. fragilis*	PRJNA206152	1	1				
80	*Bacteroides fragilis* str 3783 N1-6	*B. fragilis*	PRJNA206153	1	1				
81	*Bacteroides fragilis* str 3783 N1-8	*B. fragilis*	PRJNA206154	1	1				
82	*Bacteroides fragilis* str 3783 N2-1	*B. fragilis*	PRJNA206155	1	1				
83	*Bacteroides fragilis* str 3976 T7	*B. fragilis*	PRJNA206156	1	1				
84	*Bacteroides fragilis* str 3976 T8	*B. fragilis*	PRJNA206157	0					1
85	*Bacteroides fragilis* str 3986 T(B)10	*B. fragilis*	PRJNA206145	1			1		
86	*Bacteroides fragilis* str 3986 N(B)19	*B. fragilis*	PRJNA206120	0					1
87	*Bacteroides fragilis* str 3986 N(B)22	*B. fragilis*	PRJNA206148	1			1		
88	*Bacteroides fragilis* str 3986 N3	no call	PRJNA206147	1			1		
89	*Bacteroides fragilis* str 3986 T(B)13	no call	PRJNA206146	1			1		
90	*Bacteroides fragilis* str 3986 T(B)9	*B. fragilis*	PRJNA206118	1			1		
91	*Bacteroides fragilis* str 3988 T(B)14	*B. fragilis*	PRJNA206158	1	1				
92	*Bacteroides fragilis* str 3988 T1	*B. fragilis*	PRJNA206119	1	1				
93	*Bacteroides fragilis* str 3996 N(B) 6	*B. fragilis*	PRJNA206114	1	1				
94	*Bacteroides fragilis* str 3998 T(B)3	*B. fragilis*	PRJNA206159	0					1
95	*Bacteroides fragilis* str 3998 T(B) 4	*B. fragilis*	PRJNA206116	0					1
96	*Bacteroides fragilis* str A7 (UDC12-2)	*B. fragilis*	PRJNA206105	1			1		
97	*Bacteroides fragilis* str B1 (UDC16-1)	no call	PRJNA206104	1			1		
98	*Bacteroides fragilis* str DS-166	*B. fragilis*	PRJNA206109	1			1		
99	*Bacteroides fragilis* str DS-208	*B. fragilis*	PRJNA206107	0					
100	*Bacteroides fragilis* str DS-71	*B. fragilis*	PRJNA206108	1			1		
101	*Bacteroides fragilis* str Ds-233	*B. fragilis*	PRJNA206106	0					1
102	*Bacteroides fragilis* str I1345	*B. fragilis*	PRJNA206101	1			1		
103	*Bacteroides fragilis* str J-143-4	*B. fragilis*	PRJNA206102	2	1		1		
104	*Bacteroides fragilis* str J38-1	*B. fragilis*	PRJNA206103	1			1		
105	*Bacteroides fragilis* str Korea 419	*B. fragilis*	PRJNA206100	1			1		
106	*Bacteroides fragilis* str S13 L11	*B. fragilis*	PRJNA206121	1			1		
107	*Bacteroides fragilis* str S23L17	*B. fragilis*	PRJNA206172	1			1		
108	*Bacteroides fragilis* str S23L24	*B. fragilis*	PRJNA206173	1			1		
109	*Bacteroides fragilis* str S23 R14	no call	PRJNA206122	1			1		
110	*Bacteroides fragilis* str S24L15	*B. fragilis*	PRJNA206166	0					1
111	*Bacteroides fragilis* str S24L26	*B. fragilis*	PRJNA206167	1			1		
112	*Bacteroides fragilis* str S24L34	*B. fragilis*	PRJNA206168	1			1		
113	*Bacteroides fragilis* str S36L11	*B. fragilis*	PRJNA206170	1	1				1
114	*Bacteroides fragilis* str S36L12	*B. fragilis*	PRJNA206171	2	1		1		
115	*Bacteroides fragilis* str S36L5	*B. fragilis*	PRJNA206169	2	1		1		
116	*Bacteroides fragilis* str S38L3	*B. fragilis*	PRJNA206174	1			1		
117	*Bacteroides fragilis* str S38L5	*B. fragilis*	PRJNA206175	1			1		
118	*Bacteroides fragilis* str S6L3	*B. fragilis*	PRJNA206160	1			1		
119	*Bacteroides fragilis* str S6L5	*Chlamydia sp.*	PRJNA206161	1			1		
120	*Bacteroides fragilis* str S6L8	*B. fragilis*	PRJNA206162	1			1		
121	*Bacteroides fragilis* str S6R5	*B. fragilis*	PRJNA206163	1			1		
122	*Bacteroides fragilis* str S6R6	*B. fragilis*	PRJNA206164	2	1		1		
123	*Bacteroides fragilis* str S6R8	*B. fragilis*	PRJNA206165	1			1		
124	*Bacteroides sp.* 2 1 16	*B. fragilis*	PRJNA38347	2	1		1		
125	*Bacteroides sp.* 2 1 56FAA	*B. fragilis*	PRJNA40013	3	2		1		
126	*Bacteroides sp.* 3 2 5	*B. fragilis*	PRJNA32441	2	1		1		
127	*Bacteroides intestinalis* DSM 17393	*B. intestinalis*	PRJNA20523	0					
128	*Bacteroides massiliensis* B84634	no call	PRJNA199226	0					1
129	*Bacteroides massiliensis* dnLKV3	no call	PRJNA175977	0					
130	*Bacteroides nordii* CL02T12C05	*B. nordii*	PRJNA64823	0					
131	*Bacteroides nordii* JCM 12987	*B. nordii*	PRJNA224116	0					
132	*Bacteroides sp.* HPS0048	*B. nordii*	PRJNA72497	0					
133	*Bacteroides oleiciplenus* YIT 12058	*B. oleiciplenus*	PRJNA46377	0					
134	*Bacteroides ovatus* 3 8 47FAA	*B. ovatus*	PRJNA40011	0					
135	*Bacteroides ovatus* ATCC 8483	*B. ovatus*	PRJNA18191	0					
136	*Bacteroides ovatus* CL02T12C04	*B. ovatus*	PRJNA64825	1	1				
137	*Bacteroides ovatus* CL03T12C18	*B. ovatus*	PRJNA64827	0					
138	*Bacteroides ovatus* SD CMC 3f	*B. ovatus*	PRJNA42769	1	1				
139	*Bacteroides ovatus* str 3725 D1 iv	*B. ovatus*	PRJNA206123	0					
140	*Bacteroides ovatus* str 3725 D9 iii	*B. ovatus*	PRJNA206124	0					
141	*Bacteroides sp.* 3 1 23	*B. ovatus*	PRJNA38771	0					
142	*Bacteroides sp.* D2	*B. ovatus*	PRJNA32449	0					
143	*Bacteroides plebeius* DSM 17135	*B. plebeius*	PRJNA27829	0					
144	*Bacteroides salyersiae* CL02T12C01	*B. salyersiae*	PRJNA64829	1	1				
145	*Bacteroides salyersiae* WAL 10018	*B. salyersiae*	PRJNA170350	0					
146	*Bacteroides stercoris* ATCC 43183	*B. stercoris*	PRJNA19859	0					
147	*Bacteroides stercoris* CC31F	*B. stercoris*	PRJNA71531	1				1	
148	*Bacteroides thetaiotaomicron* VPI-5482	*B. thetaiotaomicron*	PRJNA399	0					
149	*Bacteroides thetaiotaomicron* dnLKV9	*B. thetaiotaomicron*	PRJNA175974	0					
150	*Bacteroides sp.* 1 1 14	*B. thetaiotaomicron*	PRJNA38765	0					
151	*Bacteroides sp.* 1 1 6	*B. thetaiotaomicron*	PRJNA32435	0					
152	*Bacteroides uniformis* ATCC 8492	*B. uniformis*	PRJNA18195	0					
153	*Bacteroides uniformis* CL03T00C23	*B. uniformis*	PRJNA64833	1	1				
154	*Bacteroides uniformis* CL03T12C37	*B. uniformis*	PRJNA64835	1	1				
155	*Bacteroides uniformis* dnLKV2	*B. uniformis*	PRJNA175976	0					
156	*Bacteroides uniformis* str 3978 T3 i	*B. uniformis*	PRJNA206128	0					
157	*Bacteroides uniformis* str 3978 T3 ii	*B. uniformis*	PRJNA206129	0					
158	*Bacteroides sp.* 4 1 36	*B. uniformis*	PRJNA39357	0					
159	*Bacteroides sp.* D20	*B. uniformis*	PRJNA38355	0					
160	*Bacteroides vulgatus* ATCC 8482	*B. vulgatus*	PRJNA13378	0					
161	*Bacteroides vulgatus* CL09T03C04	*B. vulgatus*	PRJNA64837	0					
162	*Bacteroides vulgatus* PC510	*B. vulgatus*	PRJNA42763	0					
163	*Bacteroides vulgatus* dnLKV7	*B. vulgatus*	PRJNA175975	1		1			
164	*Bacteroides vulgatus* str 3775 SL(B) 10 (iv)	*B. vulgatus*	PRJNA206132	1	1				
165	*Bacteroides vulgatus* str 3775 SR(B) 19	*B. vulgatus*	PRJNA206133	1	1				
166	*Bacteroides vulgatus* str 3975 RP4	*B. vulgatus*	PRJNA206134	0					
167	*Bacteroides sp.* 3 1 40A	*B. vulgatus*	PRJNA38773	0					
168	*Bacteroides sp.* 4 3 47FAA	*B. vulgatus*	PRJNA32443	1	1				
169	*Bacteroides xylanisolvens* CL03T12C04	*B. xylanisolvens*	PRJNA64839	0					
170	*Bacteroides xylanisolvens* SD CC 1b	*B. xylanisolvens*	PRJNA42773	1	1				
171	*Bacteroides xylanisolvens* SD CC 2a	*B. xylanisolvens*	PRJNA42771	1	1				
172	*Bacteroides sp.* 1 1 30	*B. xylanisolvens*	PRJNA41955	1	1				
173	*Bacteroides sp.* 2 1 22	*B. xylanisolvens*	PRJNA38349	0					
174	*Bacteroides sp.* 2 2 4	*B. xylanisolvens*	PRJNA32439	1	1				
175	*Bacteroides sp.* D1	*B. xylanisolvens*	PRJNA32447	0					
176	*Bacteroides sp.* D22	*B. xylanisolvens*	PRJNA41953	1	1				
177	*Bacteroides xylanisolvens* XB1A	no call	PRJNA39177	0					
178	*Parabacteroides distasonis* ATCC 8503	*P. distasonis*	PRJNA13485	0					
179	*Parabacteroides distasonis* CL03T12C09	*P. distasonis*	PRJNA64883	1		1			
180	*Parabacteroides distasonis* CL09T03C24	*P. distasonis*	PRJNA64885	0					
181	*Parabacteroides distasonis* str 3776 D15 i	*P. distasonis*	PRJNA206126	0					
182	*Parabacteroides distasonis* str 3776 D15 iv	*P. distasonis*	PRJNA206127	0					
183	*Parabacteroides distasonis* str 3776 Po2 i	*P. distasonis*	PRJNA206125	0					
184	*Parabacteroides distasonis* str 3999B T(B) 4	*P. distasonis*	PRJNA206130	0					1
185	*Parabacteroides distasonis* str 3999B T(B) 6	*P. distasonis*	PRJNA206131	0					2
186	*Bacteroides sp.* 2 1 33B	*P. distasonis*	PRJNA38351	0					
187	*Parabacteroides sp.* 20 3	*P. distasonis*	PRJNA38767	2		2			
188	*Parabacteroides sp.* 2 1 7	*P. distasonis*	PRJNA55579	1	1				
189	*Parabacteroides sp.* D13	*P. distasonis*	PRJNA38359	0					
190	*Parabacteroides sp.* D25	*P. distasonis*	PRJNA39405	1	1				
191	*Bacteroides sp.* 3 1 19	*P. distasonis*	PRJNA41951	0					
192	*Parabacteroides sp.* ASF519	*P. goldsteinii*	PRJNA176004	0					
193	*Parabacteroides goldsteinii* CL02T12C30	*P. goldsteinii*	PRJNA64887	0					
194	*Parabacteroides goldsteinii* dnLKV18	*P. goldsteinii*	PRJNA175978	0					
195	*Parabacteroides gordonii* DSM 23371	*P. gordonii*	PRJNA224116	1	1				
196	*Parabacteroides johnsonii* CL02T12C29	*P. johnsonii*	PRJNA64889	1	1				
197	*Parabacteroides johnsonii* DSM 18315	*P. johnsonii*	PRJNA30007	0					
198	*Parabacteroides merdae* ATCC 43184	*P. merdae*	PRJNA18193	0					
199	*Parabacteroides merdae* CL03T12C32	*P. merdae*	PRJNA64891	0					
200	*Parabacteroides merdae* CL09T00C40	*P. merdae*	PRJNA64893	0					
201	*Prevotella copri* DSM 18205	*Pr. copri*	PRJNA30025	0					
202	*Prevotella stercorea* DSM 18206	*Pr. stercorea*	PRJNA65131	0					1
203	*Bacteroides sp.* VE202-11	*C. hathewayi*	PRJNA224116	0					
204	*Bacteroides sp.* Ga6A1	no call	PRJNA224116	0					
205	*Bacteroides sp*. Ga6A2	no call	PRJNA224116	0					
				115	48	9	56	2	15

**Table 2:**
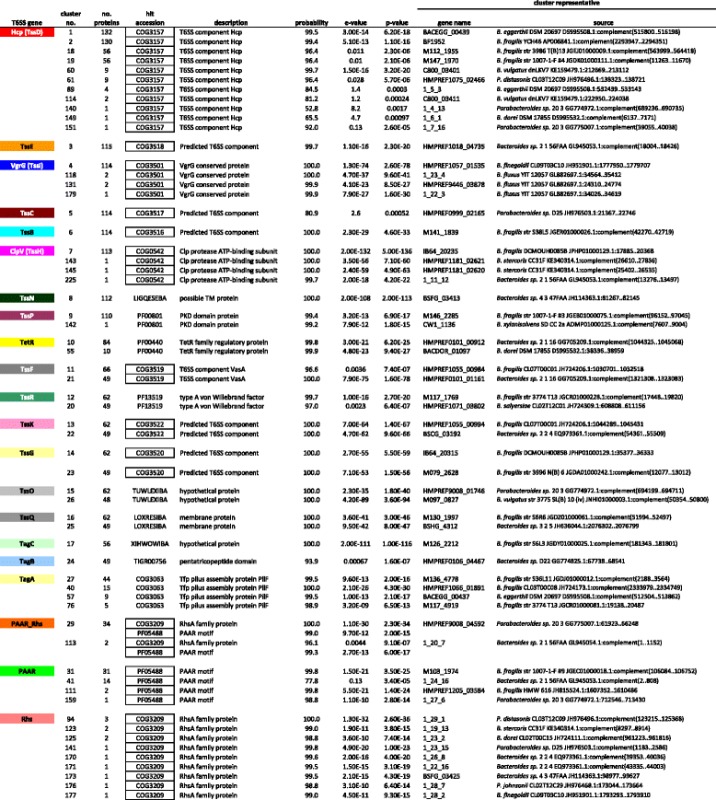
Cluster analysis of prevalent protein families of Bacteroidales T6SS loci

**Fig. 1:**
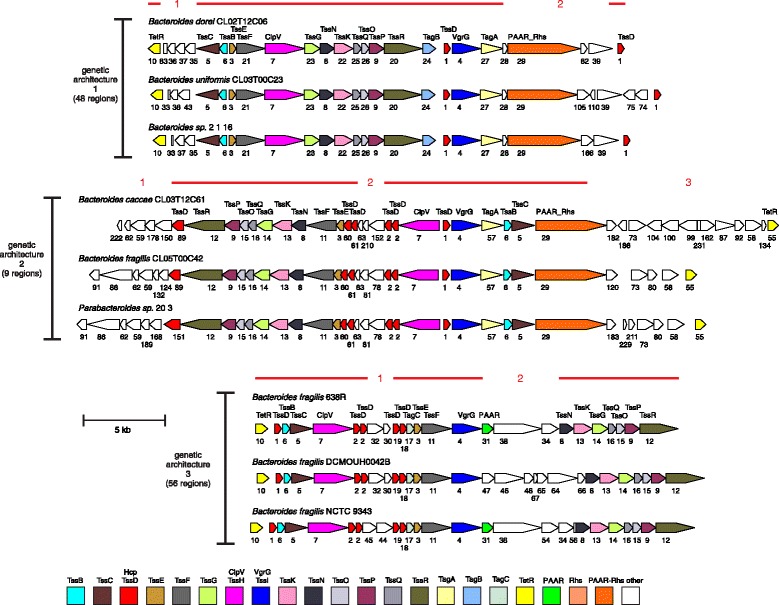
ORFmaps of three T6SS loci of each of the three genetic architectures. Genes are colored based on similarity detected by amino acid level homology, sequence-profile and profile-profile analyses, and predicted structural similarities. Regions of high DNA similarity within a genetic architecture are shown above as red lines corresponding to the strain at the top for each architecture, with the numbered breaks representing regions in which the DNA sequences diverge. Tag signifies “Type VI associated gene”

**Fig. 2:**
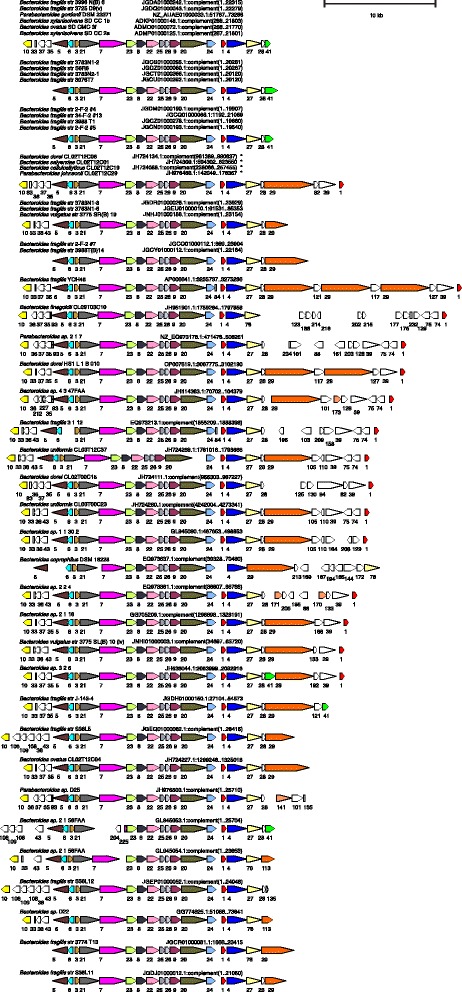
ORF maps of the human gut Bacteroidales GA1 T6SS loci. Alignment of GA1 T6SS loci demonstrating the conservation of these regions. Maps labeled with multiple isolate names indicate these DNA sequences matched each other at 99 % identity over 96 % of their lengths; an ORF map representative of each set is shown. The four co-resident isolates from the CL02 microbiome are marked by asterisks, indicating that ambiguities in these sequences were resolved by Sanger sequencing [23]. Most of the genomes are draft assemblies, and many of the segments shown comprise entire contigs -- maps that appear truncated upstream or downstream likely reflect difficulties during assembly. The number of the cluster into which the encoded protein segregated is shown under each gene. The ORF maps are colored according to the key provided in Fig. 1

**Fig. 3:**
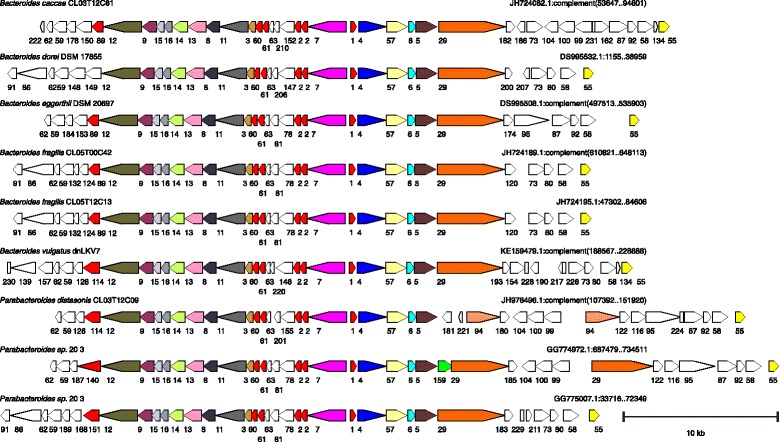
ORF maps of the human gut Bacteroidales GA2 T6SS loci. Alignment of GA2 T6SS loci demonstrating the conservation of these regions. The number of the cluster into which the encoded protein segregated is shown under each gene. The ORF maps are colored according to the key provided in Fig. 1

**Fig. 4:**
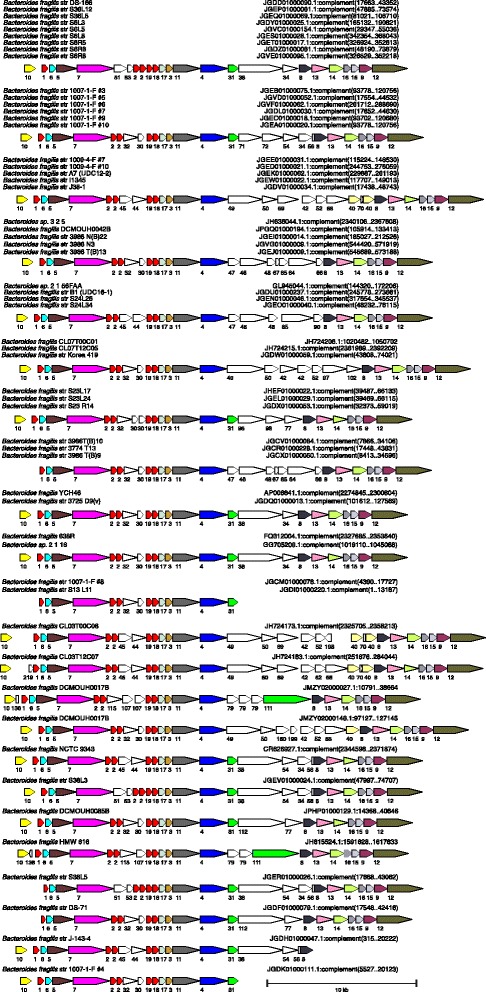
ORF maps of the human gut Bacteroidales GA3 T6SS loci. Alignment of GA2 T6SS loci demonstrating the conservation of these regions. Maps labeled with multiple isolate names indicate these DNA sequences matched each other at 99 % identity over 96 % of their lengths; an ORF map representative of each set is shown. This architecture was found in *B. fragilis* only. Most of the genomes are draft assemblies, and many of the segments shown comprise entire contigs -- maps that appear truncated upstream or downstream likely reflect difficulties during assembly. The number of the cluster into which the encoded protein segregated is shown under each gene. The ORF maps are colored according to the key provided in Fig. 1

A sequence was randomly chosen (the “cluster representative”) from the members of the cluster and used to create a profile-HMM (see text). The representative profile-HMM was used as a query against databases of profile-HMMs (profile-profile comparison). Representative hits are shown. Boxed entries in the “hit accession” column indicate motifs that have previously been considered determinative for the type of T6SS protein [12, 26, 48–51] many of these relationships are undetectable by standard sequence-profile analyses. Only proteins found in the mapped T6SS regions (see Figs. 1, 2, 3 and 4) are listed here. Gene names of the form 1_n_n indicate translations by Prodigal 2.2.6 [45] that did not match the depositor-supplied translations. The cell colors used are consistent with those used on the open reading frame maps

### *Bacteroides* and *Parabacteroides* T6SS loci segregate into three major genetic architectures

ORF maps of each of the original 115 T6SS regions were created with all genes encoding proteins of the same family designated by identical color (Figs. 1, 2, 3 and 4). All regions were oriented so that *vgrG* is transcribed left to right. Analysis of these ORF maps revealed that these T6SS loci segregate into three distinct genetic architectures (Figs. 1, 2, 3 and 4, Table 1, and Additional file 1). These genetic architectures are easily distinguished by the consistent organization and orientation of the T6SS genes. Those we are calling genetic architecture 1 (GA1) (48 regions) are found in the genomes of 13 different species, including both *Bacteroides* and *Parabacteroides*. Genetic architecture 2 (GA2) includes nine T6SS loci present in seven different *Bacteroides* and *Parabacteroides* species. Genetic architecture 3 (GA3) includes 56 loci and are present exclusively in *B. fragilis* strains. The overrepresentation of GA3 is due to the large number of *B. fragilis* genome sequences deposited in the databases (87 of the 205 strains). Only two of the 115 T6SS loci do not segregate into one of these three genetic architectures (Table 1 and Additional file 1). A strain can harbor more than one T6SS locus, as eight strains contain both a GA1 and a GA3 T6SS locus (Table 1). However, no strains were identified that contain a GA2 T6SS locus with either of the other two T6SS genetic architectures (Table 1). Alignment of the DNA of the T6SS loci within a genetic architecture revealed a high degree of DNA identity between loci, whereas little to no DNA identity exists between T6SS loci of different genetic architectures. Each genetic architecture also had breaks in these conserved regions were the DNA was divergent. The red lines above the first T6SS locus of each genetic architecture shown in Fig. 1 show the areas of highly identical DNA, with the red number in the breaks representing the divergent/variable regions. Within the conserved areas, the GA1 regions were > 95 % identical, the GA2 regions were >80 % identical, and the GA3 regions were >95 % identical between strains within a genetic architecture.

### Conserved Bacteroidales Tss proteins not present in Proteobacterial T6SSs

In Proteobacteria, there are 13 core Tss proteins [26]. By motif and profile HMM analysis, we identified presumed functional equivalents of nine of these 13 Proteobacterial core proteins in gut Bacteroidales T6SS loci; however, genes encoding identifiable TssA, TssJ, TssL and TssM proteins were not detected. The function of TssA is currently unknown; however, TssJ, TssL, and TssM likely form a transenvelope complex that anchors the phage tail structure [27, 28]. There are five proteins of unknown function encoded within the Bacteroidales T6SS of all three genetic architectures. Four of these proteins (TssN – TssQ) were previously detected in a few Bacteroidetes species [24]. The fifth protein encoded by all three T6SS genetic architectures of human gut Bacteroidales we are designating TssP. Using the HMM profiles created for these proteins, searches of all 205 genomes showed that genes encoding these proteins are contained almost exclusively in T6SS loci (Additional file 1). TssN proteins have multiple predicted transmembrane (TM) regions, whereas TssP, TssO, and TssQ proteins each have one predicted TM region near their N-termini, and TssR proteins have no predicted TM regions. It is likely that some or all of these Bacteroidetes-specificT6SS proteins perform functions analogous to those performed by TssA, TssJ, TssL, and TssM of the Proteobacterial T6SSs. In the Bacteroidales T6SS loci, these five conserved *tss* genes are adjacent to genes encoding TssG and TssK (Fig. 1). These two proteins have recently been shown in Proteobacteria to localize to the inner membrane and to interact with each other and with TssF and are predicted to form a stable subcomplex within the basal structure of the T6SS complex [29]. We have designated three proteins of unknown function with the nomenclature of Tag (Type VI associated gene) due to their conservation in at least one genetic architecture. TagA is encoded by T6SS loci of both GA1 and GA2. TagB is encoded only by T6SS loci of GA1; and TagC is encoded only by GA3 T6SS loci (Additional file 2).

### Multiple Hcp encoding genes

The 115 identified Bacteroidales T6SS loci encode 400 Hcp proteins (Additional file 2). Hcp proteins form hexamers that stack to form the inner tube/needle structure of the puncturing device [11]. Other functions have also been described for Hcp proteins, for example, some Hcp proteins have been shown to have a chaperone function in that they bind to and stabilize effectors [13]. In addition, there are “evolved” Hcp proteins that have the Hcp domain at the N-terminal half of the protein and a toxic effector function present in the C-terminal portion of the protein [30]. Our analyses showed that GA1 T6SS loci encode two Hcp proteins, both of which are contained in cluster 1 (Additional file 2), GA2 T6SS regions typically encode five distinct Hcp proteins of four different clusters and a larger “evolved” Hcp that segregate to distinct clusters based on the toxin region contained in the C-terminus (Additional file 2). GA3 T6SS loci typically encode five Hcp proteins that segregate into four clusters. Most T6SS loci of Proteobacterial species only encode a single Hcp protein and the biological relevance for the presence of multiple *hcp* genes in these Bacteroidales T6SS regions is currently unknown, but is another distinguishing feature of Bacteroidales T6SSs compared to those of Proteobacteria.

### Transcriptional regulators of the TetR/AcrR family

Type VI secretion is a highly regulated process in many species with numerous factors affecting regulation (reviewed [9, 28]). Bacteroidales T6SS loci of all three genetic architectures contain a nearby gene encoding a protein of the TetR/AcrR transcriptional regulator family. TetR proteins bind to DNA and repress transcription [31]. When its ligand is present, the ligand binds TetR and releases it from the DNA allowing for transcription. Although TetR family proteins have not to date been shown to regulate T6S, we predict, based on the vicinity of these TetR encoding genes to theT6SS genes of all three genetic architectures, that these proteins may have a role in transcriptional regulation of T6SS loci in the gut Bacteroidales. TetR orthologs from GA1 and GA3 are highly similar to each other and segregate into the same cluster (Additional file 2); whereas the GA2 TetR orthologs have almost no similarity at the protein sequence level to those of GA1 and GA3, and may regulate T6S in a different manner, and/or respond to different ligands.

### Effector and Immunity proteins

The highly identical DNA of a given T6SS genetic architecture (Fig. 1) contains genes encoding core Tss proteins and conserved Tags (Fig. 1), whereas the divergent regions do not, and some genes in these regions encode identifiable effector and/or immunity proteins (Additional file 3). Therefore, different T6SS loci of a given genetic architecture often encode different effector and immunity proteins. For GA1 T6SS loci, the DNA similarity in region 2 ends just after a gene encoding a protein of immunity family 10 followed by a gene encoding a PAAR-Rhs-effector, also described as polymorphic toxins [21]. In many of these genes, the contig ends or contains stretches of Ns due to inherent difficulties in assembling Rhs regions. However, the sequence of many of these PAAR-Rhs-effector regions is complete and some of these polymorphic toxins include C-terminal WapA domains or YwqJ nucleic acid deaminase domains (Additional file 3) [32]. GA2 T6SS loci encode the greatest number of identifiable effector/immunity proteins, all of which are contained within the three variable regions shown in Fig. 1. Identifiable effectors include “evolved” Hcp with predicted C-terminal toxin domains including DYW nucleic acid deaminase superfamily and toxin 43 superfamily predicted to have RNase activity [21], as well as many unknown toxin domains in various GA2 regions. GA2 region 2 encodes PAAR-Rhs-effector toxins, many of unknown function; although several distinct toxin domains were identified including that of the AHH nuclease family, a URI fold nuclease toxin 2 family and a colicin-like nuclease. In addition, there are other predicted effectors/immunity proteins encoded in divergent regions of some GA2 loci including a cell wall hydrolase *tae4/tai4* effector/immunity gene pair in one strain, and a pore-forming colicin-like protein. The GA3 T6SS loci have two variable regions, the genes of which are largely of unknown function. In the *B. fragilis* 638R and 9343 T6SS loci, all proteins encoded in these two divergent regions contain transmembrane spanning regions, and we predict these constitute as yet undescribed families of effector and immunity proteins.

To more comprehensively identify effector and immunity proteins in these T6SS regions, we took advantage of a comprehensive study of toxins and immunity proteins associated with polymorphic toxins (21). We created HMMs of the segment alignments for the 220 toxin and immunity proteins described in this study and compared all of the proteins encoded by the 115 T6SS loci to these HMM models. These comparisons identified numerous effector and immunity proteins (Additional file 3), most of which were encoded by GA1 and GA2 T6SS loci, which encode the majority of polymorphic toxins.

### Bacteroidales T6SS loci and ICE

**Fig. 5:**
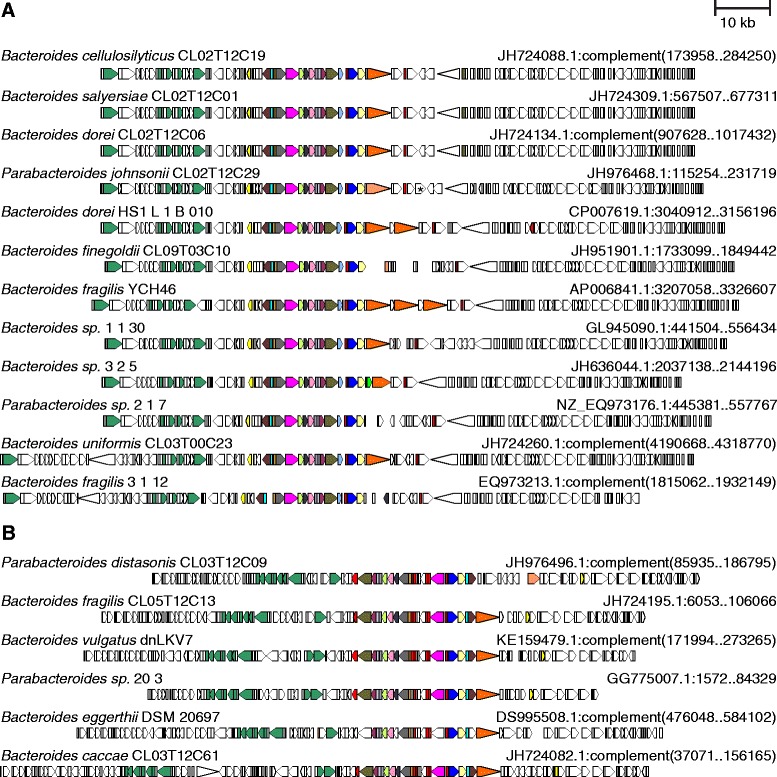
Comparison of the ICEs containing GA1 and GA2 T6SS loci. *tra* genes (dark green) are adjacent to each of the T6SS loci and present in a consistent manner within an ICE of a genetic architecture. **a** For the GA1 ICE, the *tra* genes from left to right encode TraG (TIGR03783), TraK (TIGR03781), TraM (TIGR03779), TraN (TIGR03780), TraD (TIGR02759). **b** For GA2 containing ICE, the *tra* genes from left to right encode TraO (PF10626), TraN (TIGR03780), TraM (TIGR03779), TraK (TIGR03781), TraJ (TIGR03782), TrbJ (TIGR02780), TraG (TIGR03783), TraI (PRK13878), TraD (TIGR02759). The four CL02T12 isolates from the same microbiome are drawn using sequence with ambiguities corrected. An IS element interrupting the continuity in *P. johnsonii* CL02T12C29 is marked with an asterisk [23]. The ORF maps are colored according to the key provided in Fig. 1, except that *tra* genes are additionally colored dark green

We previously showed that a 116-kb integrative conjugative element (ICE) containing a T6SS locus was transferred between five co-resident Bacteroidales species of a human subject [23]. As the transfer of these antagonistic systems has important ecological implications, we analyzed whether other T6SS loci may also be contained on ICE, and therefore, subject to intra-ecosystem transfer. The genomes of all strains containing a T6SS locus present on a sufficiently large contig were searched for genes encoding conjugative transfer (Tra) proteins, many of which we found to be consistently encoded by Bacteroidales ICE [23]. These analyses demonstrated that T6SS loci of GA1 and GA2 have *tra* genes in very close proximity to the T6SS loci (Fig. 5). In addition, the *tra* genes flanking GA1 T6SS loci are present in a consistent pattern, as are the *tra* genes flanking GA2 T6SS loci. These similarities suggest not only that the T6SS loci of a genetic architecture are contained on ICE, but they are contained on very similar ICE. To determine how similar the ICE harboring GA1 or GA2 T6SS loci are, we extended the DNA alignment analyses to these flanking regions and determined the extent of DNA similarity between strains of a given architecture. Using the defined 116 kb ICE from the CL02 strains containing the GA1 T6SS locus, we found that there was remarkable similarity among GA1-harboring strains with most strains at least 95 % identical at the DNA level along the length of the ICE, and some more than 99 % identical (Additional file 4). For GA2, there was also extensive DNA identify albeit to a lesser degree with identity values ranging from approximately 75 to 99 % along the approximately 100 kb ICE (Additional file 5). In contrast, analysis of regions flanking GA3 T6SS loci did not reveal a consistent pattern of *tra* genes in close proximity to the T6SS loci. Only three of the GA3 T6SS loci contained these *tra* genes within 50 kb of the T6SS loci, and the distances were variable between strains with some being several hundreds of kilobases away. Bacteroidales strains are known to harbor numerous conjugative elements of which these distantly encoded Tra proteins may be part. Collectively, these data strongly suggest that GA1 and GA2 T6SS loci are contained on ICE, which explains their distribution among many species and families of gut Bacteroidales. In addition, the ICE harboring GA1 are extremely similar to each other at the DNA level as are the GA2 harboring ICE. The data are less supportive of the presence of GA3 T6SS loci on ICE and may explain why these T6SSs are restricted to *B. fragilis*.

### Analysis of T6SS loci from natural human gut Bacteroidales communities

Of the 205 human gut Bacteroidales strains that were analyzed for T6SS loci, seven strains were co-resident in the gut ecosystem of human subject 2 (CL02 strains) and eight were co-resident in the gut ecosystem of human subject 3 (CL03 strains) [1]. This allowed us the unique opportunity to determine the T6SS loci profiles of strains in natural microbial communities. We previously showed that an integrative conjugative element (ICE) containing a T6SS locus was transferred between five of seven co-resident CL02 strains [23]. Our current study now reveals that this T6SS locus is a member of the GA1 group. In addition, these studies reveal that two other co-resident Bacteroidales strains that were isolated from this individual do not contain a T6SS locus. Therefore, all Bacteroidales strains isolated from this individual either have an identical GA1 T6SS region that was transferred between strains, or no T6SS locus. The situation is very different when analyzing the strains from subject 3. Our analyses reveal that of the eight CL03 strains sequenced, four contain T6SS loci, each of which are distinct. *B. uniformis* CL03T12C37 has a GA1 T6SS locus, *B. caccae* CL03T12C61 and *P. distasonis* CL03T12C09 each have GA2 T6SS loci that are distinct, and *B. fragilis* CL03T12C07 has a GA3 T6SS locus. It will be important to determine if these co-resident strains with distinct T6SS loci antagonize each other, or if there are features that allow them to peacefully co-exist. These data demonstrate that co-resident Bacteroidales strains from human gut ecosystems do not fall within a single pattern in regard to the types of T6SS loci that they harbor, rather, there are distinct patterns of T6SS loci in co-resident strains.

### Evidence of intraecosystem transfer of GA1 T6SS loci

**Fig. 6:**
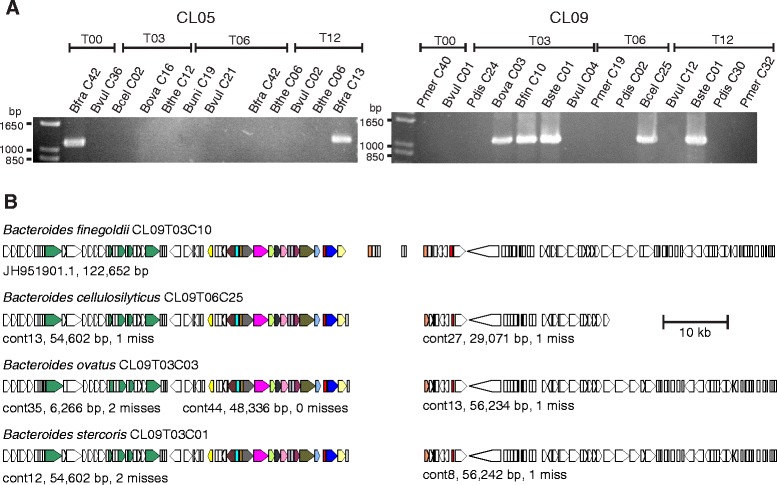
Analysis of T6SS loci transfer between co-resident strains. **a** Ethidium bromide-stained agarose gels showing the results of PCR amplification of regions specific to the *B. fragilis* CL05T12C13 GA2 T6SS locus (left) or the *B. finegoldii* CL09T03C10 GA1 T6SS locus (right) from co-resident strains. Bfra - *B. fragilis*, Bvul – *B. vulgatus*, Bcel – *B. cellulosilyticus*, Bova – *B. ovatus*, Bthe – *B. thetaiotaomicron*, Buni – *B. uniformis*, Pmer – *P. merdae*, Pdis – *P. distasonis*, Bfin – *B. finegoldii*, Bste – *B. stercoris*. The entire strain designation consists of three parts: a subject ID (e.g. CL09), an indicator of the isolation time in months (e.g. T03), and a colony ID (e.g. C10). **b** Comparison of T6SS-containing ICE DNA contained within four co-resident species. The three *Bacteroides* isolates sequenced for this work (*B. cellulosilyticus*, *B. ovatus*, and *B. stercoris*) contain DNA nearly identical to a previously sequenced isolate (*B. finegoldii*) from the same individual, strongly suggesting transfer of this ICE among co-resident strains. The small ORFs in the center of the *B. finegoldii* map are surrounded by Ns; this DNA is present in the three newly sequenced strains as well, but as separate small contigs, as the assembler used took a less aggressive scaffolding approach. The ICE containing this GA1 T6SS locus is greater than 110,000 bp in size. The ORF maps are colored according to the key provided in Fig. 1, except that *tra* genes are additionally colored dark green

Unlike the transfer of the GA1 T6SS locus ICE between co-resident CL02 strains [23], neither the GA1 nor the two GA2 T6SS loci of the CL03 strains from subject 3 are present in more than one Bacteroidales member of this ecosystem that we analyzed. To determine if we could detect additional transfers of T6SS loci via ICE, we broadened these analyses to co-resident strains from two additional human subjects. The genome sequences of two *B. fragilis* strains from subject 5 (*B. fragilis* CL05T12C13 and CL05T00C42 that are the same strain isolated at different time points) are available and these harbor a GA2 T6SS locus. The genome sequences of four strains from subject 9 are available, only one of which, *B. finegoldii* CL09T03C10, harbors a T6SS (GA1) (Table 1). We designed primers specific to these two T6SS loci (in the variable regions of each locus) and used PCR to detect the presence of these specific T6SS loci in other Bacteroidales strains from these individuals. We were unable to detect the GA2 T6SS locus in any of the other six Bacteroidales species isolated from subject 5 (Fig. 6a). However, of the seven CL09 species analyzed, PCR products specific to the GA1 T6SS locus of *B. finegoldii* CL09T03C10 were amplified from the DNA of three additional species (*B. ovatus*, *B. stercoris*, and *B. cellulosilyticus*). We sequenced the genomes of these three strains and found that they contain a nearly identical T6SS containing ICE, with most strains containing this region on two contigs with only a central portion absent from the assembled sequences (Fig. 6b). The largest difference in DNA sequence between these strains was only three mismatches, therefore, this GA1 T6SS loci containing ICE is 99.997 % identical between these four co-resident strains. Although ICE harboring GA1 T6SS loci are highly identical at the DNA level, such extremely high DNA identity does not occur between non-co-resident strains (Additional file 4 and Additional file 5). Therefore, of the four human gut Bacteroidales communities that we have analyzed, we have evidence for the transfer of GA1 T6SS via ICE among co-resident Bacteroidales strains in two of these ecosystems.

## Discussion

The last decade has witnessed an incredible expansion of our understanding of the composition of the human gut microbiota and the genomes of its microbial members. These data have transformed our ability to analyze this microbial community and to ask fundamental questions regarding the mutualistic and competitive interactions that govern its composition. Competition for nutrients, or exploitative competition, is likely a driving factor in ecosystem composition and numerous studies have been directed at understanding the nutrient utilization capabilities of various members (reviewed [5]). We previously demonstrated that interference competition also occurs among human gut Bacteroidales strains by the production of factors that directly harm other members. Bacteroidales secrete antimicrobial proteins, termed BSAPs, the first of which was shown to be secreted in OMVs [7]. The present study demonstrates that these bacteria also employ T6SSs, likely to antagonize other strains, and that these loci are widely distributed among human gut Bacteroidales strains. We have identified numerous different types of effectors in these T6SS loci likely with cellular targets including the cell membrane, peptidoglycan, and nucleic acid in addition to many with functions yet to be described.

In order to better understand the role of T6SSs in established gut communities, it will be important to analyze antagonistic interactions between co-resident bacteria isolated from human gut ecosystems. The present study has revealed two disparate situations in regards to Bacteroidales T6SS loci from two human subjects. In one ecosystem, all seven Bacteroidales strains either have an identical GA1 T6SS locus that was transferred between members via an ICE, or they completely lack a T6SS. In the second ecosystem, four Bacteroidales members each have a distinct T6SS loci and four other members have none. This finding raises the important question as to whether strains without T6SS are subject to antagonism by those equipped with these machineries, and whether the T6SS-containing strains from subject 3 are antagonistic to each other. This highlights our current limited knowledge regarding the spatiality of the Bacteroidales community. It will be important to determine if niches of co-resident strains are over-lapping, with antagonistic interactions frequently occurring, or whether these competitive interactions only occur if one member encroaches into another’s territory. There is also the question of the role of these systems in ecosystem invasion by a new strain, or in the defense of an ecosystem from invading strains. It should be possible to address these important ecological questions using well established gnotobiotic mouse models.

The T6SS loci of human gut Bacteroidales clearly segregate into three distinct genetic architectures with only a few outliers. GA1 T6SSs are extremely related to each other and appear to be readily transferred between co-resident Bacteroidales strains. The data also strongly support that GA2 T6SS loci are contained on ICE, however, many fewer of GA2 T6SS loci were identified in these gut Bacteroidales strains, indicating they may be less amenable to transfer among co-resident strains.

It is intriguing that none of the four *B. thetaiotaomicron* genomes analyzed in this study contained a T6SS locus. This is the only species for which there were four representative strains, none of which contained a T6SS locus. It is possible that as more *B. thetaiotaomicron* strains are sequenced, such regions will be identified. Alternatively, it is possible that *B. thetaiotaomicron* strains are not recipients of T6SS loci containing ICE. In contrast, a large majority of *B. fragilis* strains contain T6SS loci (75 of 87 strains), most of GA3, although GA1 were also frequently present, and both were occasionally present on the same genome. Due to the incomplete nature of many of these genomes, it is possible that even a larger percentage of *B. fragilis* strains may contain T6SS loci.

This comprehensive analysis of the T6SS loci of human gut Bacteroidales has revealed many unique features of these systems that can serve as a foundation for future investigation. From a structure/function perspective, it will be important to delineate the roles of the conserved Tags and if they are functional equivalents of the Proteobacterial Tss proteins that are “missing” from gut Bacteroidales T6SSs. In terms of regulation, it will be important to determine if TetR is a transcritptional repressor of *tss* genes and, if so, what are the ligands, environmental signals, or cues that overcome repression. This study also revealed regions of the T6SS loci that likely encode effector and immunity proteins and illustrate that the Bacteroidales may utilize previously undescribed classes of these molecules. These future molecular analyses, combined with ecological analyses of antagonistic interactions between strains from human ecosystems and in animal models, should rapidly increase our knowledge of these ubiquitous antagonistic systems of our abundant gut bacteria.

## Conclusions

This study represents an extensive and comprehensive analysis of Type VI secretion systems in the human gut Bacteroidales. As T6SS loci were found in more than half of human gut Bacteroidales strains, and Bacteroidales comprise approximately half of the total colonic bacteria in many people, it is likely that ¼ of the bacteria in the human colon contain T6SS loci. These T6SS loci were found in Bacteroidales species from three different families, and they segregate into three evolutionarily distinct genetic architectures, two of which are contained on integrative conjugative elements. We identified five new conserved core proteins that are not encoded by Proteobacterial T6SS loci and may be functional equivalents of the four Proteobacterial Tss orthologs that are absent in these Bacteroidales T6SSs. We also identified numerous distinct effector and immunity proteins and identified regions of the loci that likely encode undescribed effector and immunity proteins. In addition, we studied natural human gut Bacteroidales communities and found evidence that the ICEs bearing T6SS loci of one of the genetic architectures is readily transferred to other members of the ecosystem in the human gut. However, we found that a stable human gut ecosystem could harbor strains with numerous different T6SS loci, raising the possibility of bacterial antagonism among stable members of our gut microbiota.

## Methods

### The curated genome collection

The GenBank [33] and RefSeq [34] assembly summary reports (downloaded from ftp://ftp.ncbi.nlm.nih.gov/genomes/ASSEMBLY_REPORTS) were used to identify all genome sequences identified as *Allistipes, Bacteroides*, *Parabacteroides*, or *Prevotella* species. Where there were duplicate and identical genomes deposited in both GenBank and RefSeq, only one entry was retained. Depositor-provided protein sequence information had to be available for the genome to be further considered. This process resulted in an initial set of 246 genomes. From this set, species where the particular genome annotation indicated the isolate sequenced was not of human origin or – if such annotation was lacking – where the type strain of the species was not of human origin (e.g. *Bacteroides barnesiae* from chickens, all *Prevotella* except *Prevotella copri* and *Prevotella stercorea*) were eliminated. All *Allistipes* species retrieved were retained.

For all retained genomes, DNA identified in the genome’s Generic Feature Format (.gff) file as 16S ribosomal RNA or, where such documentation was lacking, DNA showing best-hit BLAST homology to a 16S sequence from *B. fragilis* NCTC 9343, was extracted and compared to the 16S sequence database from the Ribosomal Database Project [35] and to the refseq_rna database at NCBI to confirm the species where indicated, or to assign a putative species ID to those genomes identified by the depositor to the genus level only. The final set of genomes encompasses 205 genome sequences, representing four genera and 36 species (Table 1).

### Detection of T6SS regions

Each retained genome was scanned using the reverse position-specific blast utility rpsblast from the 64-bit Windows version of the NCBI BLAST+ suite (version 2.2.30, [36]) for proteins matching the motifs identified by Tigrfams [37] TIGR03361 (VgrG) and TIGR03345 (ClpV) position-specific scoring matrices (PSSMs) as contained in the conserved domain database (CDD, version 3.12, [38]). For a genome to be analyzed further, proteins matching these TIGRfams had to reside on the same contig, match each sentinel motif with an e-value less than or equal to 1e-03, and be within fifteen genes of each other. For genomes exhibiting such characteristics, DNA encompassing twenty-five genes flanking the outermost detected *tssH* and *vgrG* genes was retrieved from NCBI. If the contig had less than twenty-five genes either upstream or downstream of the detected *tssH* and *vgrG* genes, all available DNA was included for that flank.

### Protein clustering and cluster identification using profile-profile comparisons

All of the 5753 proteins from the resulting set of segments (*tssH*/*vgrG ±* 25 genes) were collected and grouped into clusters of proteins matching at a minimum of 30 % identity over 70 % of each of their lengths (setting b = true) using the NCBI program blastclust (version 2.2.26). A protein sequence from each of the resulting 700 clusters was pseudo-randomly selected (*via* Perl) and used for further analysis as a representative of the cluster. A multiple sequence alignment was generated for each of the 700 representative proteins by using each as a query against the profile hidden Markov model (HMM) database Uniprot20 (dated March, 2013, downloaded from ftp://toolkit.genzentrum.lmu.de/pub/HH-suite/databases/hhsuite_dbs). The MSA was generated using the HHblits [39] program using three iterations and the default e-value cutoff of 0.001. Secondary structure information predicted for the representative protein by PsiPred (version 2.6, [40]) was added to the MSA. The HHSearch program was used to generate a profile-HMM from the MSA and use it as a query against various profile-HMM databases [41], notably the Uniprot20 (March, 2013), NR20 (Aug 12, 2011), COG (Jan 14, 2015, [42]), RCSB Protein Data Bank (PDB; Feb 14, 2015, [43]), and Pfam (version 27, [44]) profile databases (all profile databases were downloaded from ftp://ftp.tuebingen.mpg.de/pub/protevo/HHsearch/databases). (Table 2). All HH-suite programs were from the 64-bit version 2.0.16, and were compiled and run under Centros Linux 7. Custom Perl scripts were employed to parse the output from these profile-profile searches; convincing relationships between the representative profile-HMM and entries in the profile-HMM databases (generally, probability > = 90 %) were used to putatively assign an identity to the representative and thus to all proteins contained within its parent cluster.

Inspection of these preliminary identification results allowed trimming of the ranges contained within the T6SS loci in a consistent manner for each genetic architecture. A comparison of the open reading frame maps thus produced displayed inexplicable differences in some of the ORFs generated from the translations deposited in GenBank, wherein some ORFs clearly present in some isolates were truncated or were missing altogether in others despite the DNA sequences being the same. Reasoning that these anomalies were likely caused by the use of various gene prediction programs by different depositors, we re-translated the DNA sequences of all the T6SS loci in a consistent manner with Prodigal (v. 2.6.2, [45]), using a training library created from *B. fragilis* NCTC 9343. The anomalies noted when relying on the deposited translations were alleviated by this procedure. The protein sequences resulting from the re-translation step were collected and re-clustered, and analyzed by profile-profile analysis as before, and putative identities were assigned based on these results.

### Detection of *tra* genes

Motifs detecting proteins with known transfer functions (Tra proteins) were extracted from the CDD database, curated based on their annotations, and used to compile a custom position-specific scoring matrix (PSSM) database. All proteins in the 205 genome set were compared to this custom database using rpsblast, and the location of detected *tra* genes was mapped to the putative T6SS loci using the gene’s name and/or contig position to determine proximity to the T6SS loci. Ultimately, genes encoding products matching TIGR02759 (TraD), TIGR03783 (TraG), PRK13878 (TraI), TIGR03782 (TraJ), TIGR03781 (TraK), TIGR03779 (TraM), TIGR03780 (TraN), PF10626 (TraO), and TIGR02780 (TrbJ) were colored in Fig. 5.

### DNA relatedness among ICE

Genomes with T6SS loci in close proximity to *tra* genes were examined for DNA-level homology. The 109,805 bp segment of DNA earlier identified as containing a T6SS loci and common to *B. cellulosilyticus* CL02T12C19, *B. dorei* CL02T12C06, *B. salyersiae* CL02T12C01, and *Parabacteroides johnsonii* CL02T12C29 (region 2 in [23]) was used as a blastn query against individual BLAST databases custom made from all contigs of each genome containing a GA1 T6SS locus. The data was retained in table format, and high scoring segment pairs (HSPs) were sorted by query start position after removal of HSPs of less than 1000 bp (Additional file 4). A similar operation was performed to identify DNA-level homology between the genomes identified as having GA2 T6SS loci, except that initial comparisons were performed using a region encompassing 50,000 bp upstream and downstream of the T6SS locus of *Bacteroides fragilis* CL05T00C42 (chosen randomly to act as the source of the query sequence). Ultimately, after several blastn iterations, the length of the query sequence was modified as the comparisons indicated the likely start and end of the homologous region (Additional file 5).

### Generation of profile HMMs

Proteins from all re-translated T6SS regions were collected according to assigned type (TssB, TssC, etc.). Each collection was made non-redundant at 100 % identity over 100 % length, using blastclust and the non-redundant collection was aligned with Clustal Omega [46]. Each alignment was used to generate a profile HMM using hmmbuild from the HMMer 3.1b1 suite (hmmer.org; the HMMer suite programs were run under Cygwin using version 6.1 of the 64-bit dynamically-linked library). Each resulting profile HMM was then used to scan (*via* hmmsearch) the set of proteins used to generate the Clustal alignment, and the highest full sequence or best domain score was recorded and used as a threshold score during subsequent analyses using the profile HMM. These profiles and information used to generate them has been submitted to the Pfam database.

### Identification of putative toxin and immunity encoding genes described by Zhang et al. (21)

The segment alignments for 220 toxin and immunity proteins were retrieved from the supplemental files provided as part of Zhang, D., et al. [21]. A hidden Markov model of each alignment was generated using hmmbuild. These models were concatenated, and a binary representation of the models was created using hmmpress. All of the proteins encoded by the 115 T6SS loci were compared to the binary HMM models using hmmsearch. Comparison included all Prodigal retranslated proteins if they were in a T6SS loci. All matches achieving a full length e-value of less than or equal to 1 × 10^−3^ were retained and shown in Additional file 3.

### PCR analyses for evidence of intra-ecosystem transfer of ICE

PCRs were performed with Taq MasterMix (NEB) using the manufacturer’s recommendations with an annealing temperature of 59 °C. Specific amplification of GA2 of *B. fragilis* CL05T12C13 was performed with primers GTCACCAGGGATTATCAAAAGG and CACATATATAATGCATATCCCTTAGCC and specific amplification of GA1 of *B. finegoldii* CL09T03C10 was performed with primers TTCGGGTGACATGGAAGAGC and GGCGTTTCCTGTCAACATTG.

### Sequencing and assembly of additional *Bacteroides* genomes from the CL09 ecosystem

Chromosomal DNA from *B. ovatus* CL09T03C03, *B. stercoris* CL09T03C01, and *B. cellulosilyticus* CL09T06C25 was fragmented using the Covaris S2 instrument, and analyzed for fragment distribution with a Hi-Sensitivity D1K TapeStation machine, and for sufficient quantity by an SYBR qPCR assay. The DNA was sequenced using an Illumina MiSeq sequencer, producing paired-end reads of 150 bp. Assembly of the genome was performed *de novo* using Velvet 1.2.10 [47], with a k-value of 71 (determined by Velvet Optimizer 2.2.5). (http://www.vicbioinformatics.com/software.velvetoptimiser.shtml). The average depth of coverage ((no. reads used × read length)/total bases assembled) was 70X, 140X, and 127X, for the *B. ovatus*, *B. stercoris*, and *B. cellulosilyticus*, respectively. The draft genome sequences of *Bacteroides cellulosilyticus* CL09T06C25, *Bacteroides stercoris* CL09T03C01, and *Bacteroides ovatus* CL09T03C03 have been deposited in GenBank under BioProject IDs [GenBank:PRJNA283626], [GenBank:PRJNA283624], and [GenBank:PRJNA283619], respectively.

## Additional files

Additional file 1:
**Proteins encoded by each of the 205 genomes that match the profile HMMs for Tss, Tag, TetR, Rhs, PAAR and PAAR-Rhs proteins.** (XLSX 359 kb) Additional file 2:
**Enumeration of Bacterodales T6SS proteins by genetic architecture and cluster.** (XLSX 12 kb) Additional file 3:
**Identification of putative effector and immunity proteins by comparison to those identified in Zhang et al. [** 21 **].** (XLSX 22 kb) Additional file 4:
**Alignment of the ICE from the Bacteroidales CL02T12 strains against contigs of strains harboring GA1 T6SS loci.** (XLSX 48 kb) Additional file 5:
**Alignment of contigs harboring GA2 T6SS loci with the** ***B. fragilis*** **CL05T00C05 contig bearing the GA2 T6SS locus.** (XLSX 16 kb)

## Abbreviations

COG: Cluster of orthologous genes
GA: Genetic architecture
ICE: Integrative and conjugative element
Rhs: Rearrangement hot spot (gene, protein)
T6SS: Type VI secretion system
*tag*: Type VI associated gene
TM: Transmembrane
